# Reasons patients cite to their health-care professional for not initiating or completing human papillomavirus vaccination

**DOI:** 10.1093/jncics/pkad047

**Published:** 2023-07-21

**Authors:** Onyema Greg Chido-Amajuoyi, Ikponmwosa Osaghae, Sanjay Shete

**Affiliations:** Department of Epidemiology, The University of Texas MD Anderson Cancer Center, Houston, TX, USA; Department of Epidemiology, The University of Texas MD Anderson Cancer Center, Houston, TX, USA; Department of Epidemiology, The University of Texas MD Anderson Cancer Center, Houston, TX, USA; Department of Biostatistics, The University of Texas MD Anderson Cancer Center, Houston, TX, USA; Division of Cancer Prevention and Population Sciences, The University of Texas MD Anderson Cancer Center, Houston, TX, USA

## Abstract

**Background:**

Vaccination against human papillomavirus (HPV) is critical to the prevention of HPV-associated cancers. This study aimed to describe the reasons patients cited for not initiating or completing the HPV vaccination series, as reported by health-care professionals.

**Methods:**

Study data were obtained from a University of Texas MD Anderson Cancer Center population-based cross-sectional survey of health-care professionals practicing in Texas. Prevalence estimates of reasons cited for not initiating or completing HPV vaccination were estimated by patient population (parents of children and adult patients).

**Results:**

The study included 973 primary care clinicians, of whom 45.53% were physicians and 54.47% were midlevel care professionals. For parents who did not initiate HPV vaccination for their child, the most commonly cited reasons were the belief that the vaccine was not needed (52.54%, 95% CI = 48.90% to 56.15%), that the child was not sexually active (52.54%, 95% CI = 48.90% to 56.15%), and safety concerns/side effects (47.05%, 95% CI = 43.44% to 50.69%). Among age-eligible adults who did not initiate HPV vaccination, lack of knowledge and awareness was the most commonly cited reason (30.52%, 95% CI = 27.71% to 33.50%). For noncompletion of the HPV vaccine series, parents most commonly cited competing priorities (41.29%, 95% CI = 37.76% to 44.91%), followed by adverse reactions after the first dose (16.05%, 95% CI = 13.56% to 18.90%). Similarly, for noncompletion of the HPV vaccine series among adults, competing priorities was the most cited reason (31.04%, 95% CI = 28.20% to 34.02%).

**Conclusion:**

These findings highlight the importance of addressing misconceptions and improving education about HPV vaccination to increase vaccination uptake rates and prevent HPV-related cancers.

Human papillomavirus (HPV) is a sexually transmitted infection associated with several cancers, including cervical, anal, penile, and oropharyngeal cancers (1). HPV vaccination is a safe and effective way to prevent these types of cancers, but not all individuals initiate or complete the vaccination series (2). Patients cite various reasons to health-care professionals for their decision regarding vaccination.

Several studies have investigated the reasons parents and patients cite for not initiating or completing HPV vaccination (3-5). Commonly cited reasons range from the belief that the child is not sexually active, perceived low risk of HPV infection, concerns about the safety and efficacy of the vaccine (3,6), lack of knowledge and awareness of HPV and the HPV vaccine (5), interpersonal factors, societal factors, and access to health care. Citation of concerns about side effects and the safety of the HPV vaccine, however, has been shown to have a steady upward trend over a 10-year period compared with other reasons for nonvaccination (3).

The state of Texas has one of the lowest HPV vaccination rates in the United States (7), which makes it an important case study for barriers to HPV vaccination initiation and uptake. Insights into the reasons patients cite to their health-care professional for not initiating or completing the HPV vaccination series are important for developing effective strategies to increase vaccine uptake. The perspective of health-care professionals who interact with parents and patients about HPV vaccination is crucial, but to the best of our knowledge, this factor has not previously been examined. Hence, this study analyzed a state-wide survey of health-care professionals with the aim of describing the reasons parents and patients cite to their health-care professionals for not initiating or failing to complete HPV vaccination.

## Methods

### Study population

The study data were obtained from a population-based cross-sectional survey developed at the University of Texas MD Anderson Cancer Center and conducted between January and April 2021. The target population for the survey included all health-care professionals currently practicing in Texas. Health-care professionals were defined as physicians with an MD or equivalent degree in the specialties of internal medicine, family medicine, obstetrics/gynecology, and pediatrics as well as physician assistants and nurse practitioners. A list of all health-care professionals currently practicing in Texas at the time of the study was obtained from the LexisNexis master referential database under an annual license. The database also contains the email address and a few demographic characteristics of these professionals, including their sex and practice type (eg, family physician, pediatrician, internal medicine, nurse practitioner, physician assistant). All eligible health-care professionals were sent an email invitation to complete an online health-care professional survey. All health-care professionals provided informed consent. A total of 1283 health-care professionals completed the study, accounting for a response rate of 7%. There was no statistically significant difference between respondents and nonrespondents regarding health-care professional sex and practice type, the 2 variables for which data were available for nonrespondents in the LexisNexis database. The study was approved by the MD Anderson Cancer Center Ethical Review Board (IRB No. 2019-1257) and followed the Strengthening the Reporting of Observational Studies in Epidemiology guidelines to ensure the ethical and scientific integrity of the research.

### Measures

Health-care professionals were identified based on the patient population they serve, which allowed us to assess study measures for 2 distinct patient populations: 1) parents of children eligible for HPV vaccination and 2) adult (aged ≥18 years) patients. Health-care professionals who indicated that they serve “only children” or “children and adults” were then asked to select all that applied from a set of possible reasons parents cited for 1) not initiating HPV vaccination, using the survey question “Which reasons do PARENTS cite for NOT initiating HPV vaccination in their child?” and 2) not completing the HPV vaccination series, using the survey question “Which reasons are cited by PARENTS for NOT completing recommended HPV vaccination series for their children?” See the survey questionnaire in the Supplementary Material (available online). Health-care professionals who indicated that they serve “children and adults” or “only adults” were then asked to select all that applied from a set of possible reasons that adult patients cite for 1) not initiating HPV vaccination, using the survey question “Which reasons do ADULT PATIENTS (>18 years) cite for NOT initiating HPV vaccination?” and 2) not completing the HPV vaccination series, using the survey question “Which reasons are cited by ADULT PATIENTS (>18 years) for NOT completing recommended HPV vaccination series?”

### Data analysis

Our analysis was restricted to health-care professionals practicing in facilities that offer HPV vaccination to age-eligible patients (children and adults older than 18 years of age). The baseline characteristics of health-care professionals in our study were estimated using survey frequencies. Estimates were stratified by patient population (parents of children and adult patients) and by HPV vaccination initiation and completion. Furthermore, estimates were stratified by health-care professional characteristics. Prevalence estimates of reasons cited for not initiating or completing HPV vaccination were estimated with corresponding 95% confidence intervals (CIs). All analyses were conducted using Stata/IC, version 15.1, statistical software (StataCorp LLC, College Station, TX).

## Results

The study comprised 973 primary care clinicians, of whom 443 (45.53%) were physicians and 530 (54.47) were mid-level care professionals, with 103 (10.79%) younger than 35 years of age, 589 (61.68%) 35 to 54 years of age, and 263 (27.54%) 55 years of age or older. Of the total number of health-care professionals, 282 (28.98%) worked in university/teaching hospitals, 325 (33.40%) were in group practices, and 120 (12.33%) were in in federally qualified health centers or public facilities (Table 1).

**Table 1.** Baseline descriptive statistics of health-care professionals in Texas^a^

Characteristic	Overall (N = 973)
Clinician age, No. (%), y	
<35	103 (10.79)
35-54	589 (61.68)
≥55	263 (27.54)
Sex, No. (%)	
Female	732 (76.49)
Male	225 (23.51)
Race and ethnicity, No. (%)	
Hispanic	138 (14.53)
Non-Hispanic Black	94 (9.89)
Non-Hispanic Other	235 (24.74)
Non-Hispanic White	483 (50.84)
Practice location, No. (%)	
Rural	41 (4.22)
Urban	931 (95.78)
Clinician type, No. (%)	
Nonphysician	530 (54.47)
Physician	443 (45.53)
Type of practice, No. (%)	
University/teaching hospital	282 (28.98)
Solo practice	96 (9.87)
Group practice	325 (33.40)
Federally qualified health center or public facility	120 (12.33)
Other	150 (15.42)
Patients seen/wk, No. (%)	
≤50	431 (45.32)
51-100	407 (42.80)
>100	113 (11.88)

a 18 observations missing for age, 16 observations missing for sex, 23 observations missing for race and ethnicity, 1 observation missing for region, and 22 observations missing for the number of patients seen per week.

### Reasons parents cited for not initiating HPV vaccination in their child

**Figure 1.**
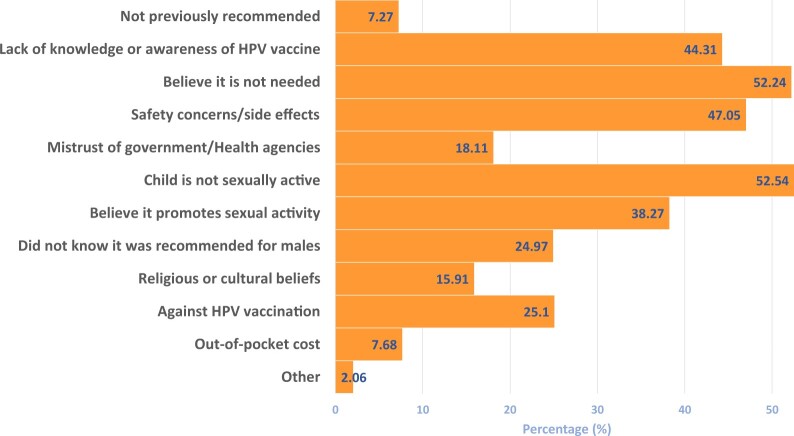
**Reasons parents have cited for not initiating HPV vaccination in their child (n = 729).** Responses are based on the survey question “Which reasons do PARENTS cite for NOT initiating HPV vaccination in their child? (Select all that apply.)” HPV = human papillomavirus.

Results revealed that the most commonly cited reasons were the beliefs that their child was not sexually active (52.54%, 95% CI = 48.90% to 56.15%) and that the vaccine was not needed (52.54%, 95% CI = 48.90% to 56.15%). Safety concerns and side effects were also commonly cited, at 47.05% (95% CI = 43.44% to 50.69%), whereas lack of knowledge and awareness about the HPV vaccine was cited by 44.31% (95% CI = 40.73% to 47.94%) of parents (Figure 1).

### Reasons adult patients cited for not initiating HPV vaccination

**Figure 2.**
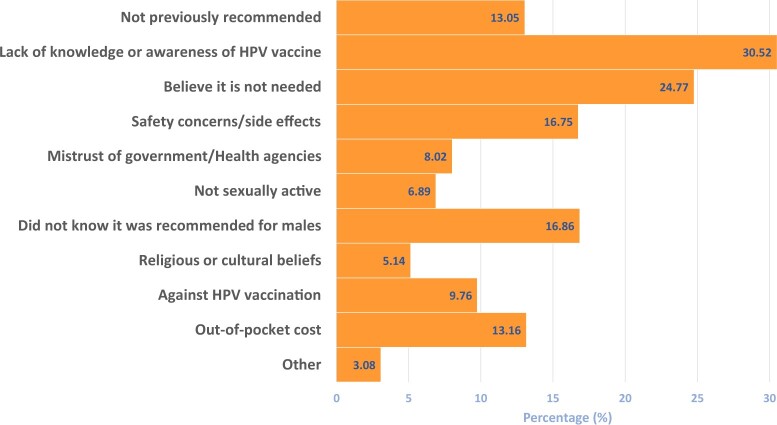
**Reasons adult patients (>18 years of age) cite for not initiating HPV vaccination (n = 973).** Responses are based on the survey question “Which reasons do ADULT PATIENTS (>18 years) cite for NOT initiating HPV vaccination? (Select all that apply.)” HPV = human papillomavirus.

Among age-eligible adults who did not initiate HPV vaccination, lack of knowledge and awareness was the most commonly cited reason, at 30.52% (95% CI = 27.71% to 33.50%), followed by the belief that the vaccine was not needed, at 24.77% (95% CI = 22.15% to 27.58%) (Figure 2).

### Reasons parents cited for not completing the HPV vaccination series in their child

**Figure 3.**
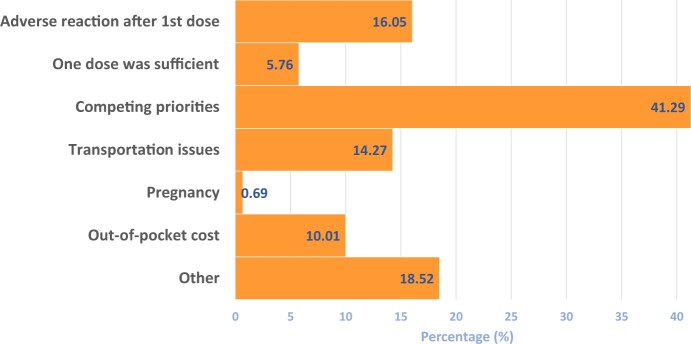
**Reasons parents cite for not completing recommended HPV vaccination series for their children (n = 729).** Responses are based on the survey question “Which reasons are cited by PARENTS for NOT completing recommended HPV vaccination series for their children? (Select all that apply.)” HPV = human papillomavirus.

For parents who did not complete the HPV vaccine series for their child, competing priorities was the most commonly cited reason, at 41.29% (95% CI = 37.76% to 44.91%), followed by adverse reactions after the first dose, at 16.05% (95% CI = 13.56% to 18.90%) (Figure 3).

### Reasons adults cited for not completing the HPV vaccination series

**Figure 4.**
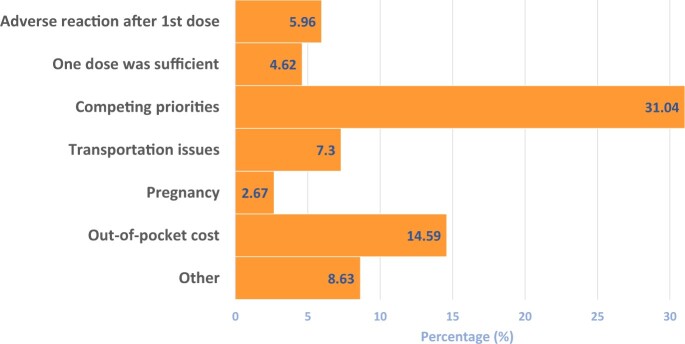
**Reasons adult patients (>18 years of age) cite for not completing recommended HPV vaccination series (n = 973).** Responses are based on the survey question “Which reasons are cited by ADULT PATIENTS (>18 years) for NOT completing recommended HPV vaccination series? (Select all that apply.)” HPV = human papillomavirus.

Among age-eligible adults who did not complete the HPV vaccine series, competing priorities were the most common reason cited, at 31.04% (95% CI = 28.20% to 34.02%), followed by out-of-pocket cost, at 14.59% (95% CI = 12.51% to 16.96%) (Figure 4).

Finally, reasons that adults and parents cited for not initiating and completing the vaccination series in their child stratified by health-care professional sex, age, and practice type are provided in Supplementary Table 1 (available online).

## Discussion

Study results revealed that cited reasons varied by HPV vaccination initiation and completion as well as by whether the respondent was a parent or age-eligible adult patient. The results of this study indicate that lack of knowledge and awareness about the HPV vaccine and the perception that the vaccine is not needed were the most common reasons cited for not initiating the vaccination series among age-eligible adults. These findings are consistent with previous studies, which have identified low knowledge and awareness of the HPV vaccine as a major barrier to vaccine uptake (4,5). More so, a study demonstrating stagnation of awareness of HPV and the HPV vaccine in the United States (8) may further exacerbate this concern among patients (4). The perception that HPV vaccination is not needed may stem from the perceived low risk of HPV (9). This finding is concerning because it overlooks the health benefits of HPV vaccination, despite strong evidence showing that the vaccine is effective in reducing the incidence of HPV-related cancers (1).

Study findings also revealed that sexual activity status and concerns about safety and side effects were most commonly cited by parents as reasons for not initiating the HPV vaccination series. At the national level, safety concerns/side effects is consistently the leading reason for HPV nonvaccination; however, as shown by our study findings, misperceptions about the interaction between sexual activity and HPV vaccination persist in Texas (3). The citation of sexual activity status as a reason for nonvaccination is consistent with previous studies (4,10) and stems from the misconception that HPV vaccination is for sexually active individuals or that it may lead to sexual disinhibition (10). Therefore, addressing this misconception and providing education on the importance of HPV vaccination before sexual activity may increase vaccine uptake.

Competing priorities were the most commonly cited reason for not completing the HPV vaccination series among parents and age-eligible adults. This finding may stem from the HPV vaccine series requiring multiple doses, which can be difficult for families to complete because of scheduling conflicts or other barriers. Single-dose HPV vaccination, which has been shown to be efficacious (11), could eliminate this barrier. Ultimately, this finding highlights the need for health-care professionals to prioritize emphasizing the importance of completing the HPV vaccination series during routine visits and addressing any barriers to that completion.

This study is not without limitations. First, health-care professional biases, such as their philosophical ideologies (eg, perceptions of vaccines) could influence their response to the survey. Similarly, consistent with most surveys of health-care professionals (12), the response rate was somewhat low, which may render our study prone to nonresponse bias. This potential was evaluated at analysis, however, and we found no statistically significant difference between respondents and nonrespondents. Finally, our study was prone to recall bias given that survey responses rely on health-care professionals’ ability to recall past events. This study provides valuable insights, however, into vaccination behaviors from those clinicians who are key stakeholders and at the other end of the table in conversations about HPV vaccination. Also, our study findings are generalizable to other states with demographics similar to our study population. Furthermore, data obtained from health-care professional surveys are robust because the responses from a single clinician often represent multiple clinician-patient encounters, providing a broader perspective on the topic.

Overall, the findings of this study underscore the importance of addressing the barriers to HPV vaccine uptake, particularly low knowledge and awareness about the vaccine and safety concerns. Through targeted education and outreach efforts, health-care professionals can be adequately equipped to address misconceptions and concerns about the HPV vaccine and promote the importance of HPV vaccination both for children and for adults. Addressing safety concerns through education and encouraging open communication with health-care professionals may alleviate vaccine hesitancy.

## Supplementary Material

pkad047_Supplementary_Data

## Data Availability

The data related to this manuscript can be obtained from the corresponding author.
