# Supplementary material for: Reasons patients cite to their health-care professional for not initiating or completing human papillomavirus vaccination
Source: JNCI Cancer Spectr. 2023 Jul 21;7(4):pkad047. doi: 10.1093/jncics/pkad047 (PMC10423071; doi:10.1093/jncics/pkad047)
Supplement: pkad047_Supplementary_Data [file pkad047_supplementary_data.pdf]

Supplementary Table 1: Reasons cited by adults and parents for not initiating and completing vaccination series in their child stratified by HCP's sex, age, provider type

	Percentage (%)	
**Reasons cited by parents for not initiating HPV vaccination in their child stratified by HCP's sex (N=720)**		
**Reasons cited**	**Male HCP (n=179)**	**Female HCP (n =541)**
Not previously recommended	10.06	6.10
Lack of knowledge or awareness of HPV vaccine	46.37	43.81
Believe it is not needed	53.63	52.87
Safety concerns / side effects	44.13	48.43
Mistrust of government / health agencies	23.46	16.45
Child is not sexually active	51.40	53.42
Believe it promotes sexual activity	37.43	39.00
Did not know it was recommended for males	25.70	24.77
Religious or cultural beliefs	15.64	15.90
Against HPV vaccination	24.02	25.69
Out-of-pocket cost	9.50	7.21
Other	1.12	2.40
**Reasons cited by adult patients (≥18 years) for not initiating HPV vaccination stratified by HCP's sex (N=957)**		
**Reasons cited**	**Male HCP (n=225)**	**Female HCP (n =732)**
Not previously recommended	14.67	12.43
Lack of knowledge or awareness of HPV vaccine	29.78	31.28
Believe it is not needed	27.56	24.18
Safety concerns / side effects	15.56	17.35
Mistrust of government / health agencies	9.33	7.65
Child is not sexually active	9.78	6.01
Did not know it was recommended for males	19.11	16.53
Religious or cultural beliefs	5.78	4.92
Against HPV vaccination	9.33	9.97
Out-of-pocket cost	16.00	12.30
Other	2.67	3.14

Reasons cited by parents for not initiating HPV vaccination in their child stratified by HCP's age (N=721)			
**Reasons cited**	**< 35 years (n=79)**	**35-54 years (n=446)**	**≥ 55 years (n=196)**
Not previously recommended	8.86	6.95	7.14
Lack of knowledge or awareness of HPV vaccine	51.90	46.41	36.73
Believe it is not needed	53.16	55.83	45.92
Safety concerns / side effects	35.44	50.90	43.88
Mistrust of government / health agencies	22.78	18.61	15.31
Child is not sexually active	46.84	53.36	54.08
Believe it promotes sexual activity	41.77	35.65	43.88
Did not know it was recommended for males	18.99	24.66	28.06
Religious or cultural beliefs	10.13	15.02	20.41
Against HPV vaccination	17.72	26.46	25.00
Out-of-pocket cost	10.13	6.28	10.20
Other	1.27	1.57	3.06
**Reasons cited by adult patients (≥18 years) for not initiating HPV vaccination stratified by HCP's age (N=955)**			
**Reasons cited**	**< 35 years (n=103)**	**35-54 years (589)**	**≥ 55 years (263)**
Not previously recommended	17.48	13.07	11.03
Lack of knowledge or awareness of HPV vaccine	37.86	29.71	29.66
Believe it is not needed	28.16	26.15	20.53
Safety concerns / side effects	17.48	16.47	17.87
Mistrust of government / health agencies	10.68	6.45	10.27
Child is not sexually active	7.77	5.60	9.51
Did not know it was recommended for males	17.48	15.11	20.91
Religious or cultural beliefs	4.85	4.75	6.08
Against HPV vaccination	12.62	9.17	9.89
Out-of-pocket cost	9.71	12.73	15.59
Other	3.88	3.06	2.66
**Reasons cited by parents for not initiating HPV vaccination in their child stratified by provider type (N=729)**			
**Reasons cited**	**Non-physicians (n=316)**	**Physicians (n =413)**	
Not previously recommended	5.06	8.96	

Lack of knowledge or awareness of HPV vaccine	45.89	43.10
Believe it is not needed	44.62	58.60
Safety concerns / side effects	43.04	50.12
Mistrust of government / health agencies	17.72	18.40
Child is not sexually active	45.57	57.87
Believe it promotes sexual activity	36.08	39.95
Did not know it was recommended for males	21.84	27.36
Religious or cultural beliefs	13.61	17.68
Against HPV vaccination	19.62	29.30
Out-of-pocket cost	8.86	6.78
Other	2.85	1.45
**Reasons cited by adult patients for not initiating HPV vaccination stratified by provider type (N=973)**		
**Reasons cited**	**Non-physicians (n=530)**	**Physicians (n =443)**
Not previously recommended	12.45	13.77
Lack of knowledge or awareness of HPV vaccine	30.94	30.02
Believe it is not needed	22.64	27.31
Safety concerns / side effects	16.42	17.16
Mistrust of government / health agencies	7.36	8.80
Child is not sexually active	6.23	7.67
Did not know it was recommended for males	16.60	17.16
Religious or cultural beliefs	4.15	6.32
Against HPV vaccination	8.30	11.51
Out-of-pocket cost	12.26	14.22
Other	3.02	3.16
**Reasons cited by parents for not recommending HPV vaccination in their child stratified by HCP's sex (N=720)**		
**Reasons cited**	**Male HCP (n=179)**	**Female HCP (n =541)**
Adverse reaction after 1st dose	20.11	14.97
One dose was sufficient	8.94	4.62
Competing priorities	37.99	42.14
Transportation issues	16.20	13.86

Pregnancy	1.68	0.37	
Out-of-pocket cost	14.53	8.50	
Other	13.41	20.33	
**Reasons cited by parents and adult patients for not recommending HPV vaccination stratified by HCP's sex (N=957)**			
**Reasons cited**	**Male HCP (n=225)**	**Female HCP (n =732)**	
Adverse reaction after 1st dose	7.56	5.60	
One dose was sufficient	5.33	4.37	
Competing priorities	30.67	31.28	
Transportation issues	8.89	6.97	
Pregnancy	2.22	2.73	
Out-of-pocket cost	16.89	13.93	
Other	5.78	9.56	
**Reasons cited by parents for not recommending HPV vaccination in their child stratified by HCP's age (N=721)**			
**Reasons cited**	**< 35 years (n=79)**	**35-54 years (446)**	**≥ 55 years (196)**
Adverse reaction after 1st dose	10.13	16.59	16.84
One dose was sufficient	3.80	5.61	7.14
Competing priorities	40.51	43.72	35.71
Transportation issues	18.99	13.68	13.78
Pregnancy	0.00	0.67	0.51
Out-of-pocket cost	12.67	7.62	14.29
Other	13.92	19.96	17.86
**Reasons cited by adult patients for not recommending HPV vaccination stratified by HCP's age (N=955)**			
**Reasons cited**	**< 35 years (n=103)**	**35-54 years (589)**	**≥ 55 years (263)**
Adverse reaction after 1st dose	4.85	5.77	6.46
One dose was sufficient	6.80	4.58	3.42
Competing priorities	36.89	32.77	25.10
Transportation issues	9.71	7.47	6.46
Pregnancy	1.94	3.40	1.14
Out-of-pocket cost	11.65	13.58	17.87
Other	8.74	7.98	10.27

Reasons cited by parents for not recommending HPV vaccination in their child stratified by provider type (N=729)		
**Reasons cited**	**Non-physicians (n=316)**	**Physicians (n =413)**
Adverse reaction after 1st dose	14.24	17.43
One dose was sufficient	6.01	5.57
Competing priorities	38.29	43.58
Transportation issues	16.14	12.83
Pregnancy	0.63	0.73
Out-of-pocket cost	12.03	8.47
Other	14.56	21.55
**Reasons cited by adult patients for not recommending HPV vaccination stratified by provider type (N=973)**		
**Reasons cited**	**Non-physicians (n=530)**	**Physicians (n =443)**
Adverse reaction after 1st dose	6.42	5.42
One dose was sufficient	5.09	4.06
Competing priorities	28.11	34.54
Transportation issues	7.55	7.00
Pregnancy	1.89	3.61
Out-of-pocket cost	14.53	14.67
Other	8.11	9.26

HPV Survey

Please complete the survey below.

Thank you!

HPV Vaccination: Healthcare Professionals'
Practices and Perceptions

Completion time is estimated to be 10 minutes or less.

Authorization for Participation in Research

The State of Texas currently ranks 41st in HPV vaccination rates in the US. The goal of this research study is to assess health care professionals' practices and perceptions regarding barriers to HPV vaccination initiation and completion in the State of Texas.

The survey has been developed by The University of Texas MD Anderson Cancer Center.

If you agree to complete this study, your personal identifiable information will be password protected and will not be directly associated with your study answers. All study data will be reported in aggregate, and no individual participant's answers will be singled out in publication or presentation.

As compensation for your time and effort, you will receive a $10 online gift card (e.g. Amazon or similar). After completing the survey, your name will also be entered into a drawing for a $1000 Amazon online gift card. The drawing will be held 3 months after the survey is closed. Your odds of winning are about 1 in 13,000. If you are the winner, you will be notified by email. If there is no response from you, two (2) follow-up emails will be sent. If there is still no response, the gift-card will be offered to the next person whose name is drawn.

Consent Statement
You have read the description of the study and have decided to participate in the research project described here. You understand that you may refuse to answer any (or all) of the questions at this or any other time. You understand that you are free to refuse any further participation if you wish.

You may be contacted in the future with regard to this study, but you are free to refuse further participation.

You may withdraw your authorization at any time. You can learn more about how to withdraw your authorization by calling 713-792-6477 or by contacting the study chair, Dr. Shete at 713-745-2483.

This section is about you and your medical practice

Which best describes your role?

◯ Family Physician
◯ Pediatrician
◯ Gynecologist
◯ Internal Medicine
◯ Physician Assistant
◯ Nurse Practitioner/Advanced Nurse Practitioner
◯ Other

Please specify your role.

____

Which best describes your workplace?

◯ Solo practice
◯ Group practice (single or multi-specialty)
◯ University or teaching hospital or affiliated clinic
◯ Faith based hospital/clinic
◯ Employed Physician Practices
◯ Federally Qualified Health Center (FQHC)
◯ City/County/Public Health Care facility
◯ Other

Please specify your Other practice.

____

Whom does your practice serve?

◯ Only children
◯ Both adults and children
◯ Only adults

The following section is about HPV vaccination.

Have you received formal training in HPV vaccination promotion or counseling (for example: continuing medical education, workshops, certified training seminars)?

◯ Yes
◯ No

How long ago did you attend a formal training session on HPV vaccination?

◯ Less than a year ago
◯ In the last 1-2 years
◯ More than 2 years ago but less than 5 years
◯ More than 5 years ago

Does your practice offer HPV vaccination?

◯ Yes, always offered
◯ Yes, but not consistently
◯ No
◯ I don't know

Please indicate why your practice setting does not consistently offer HPV vaccination. (Select all that apply)

☐ Limited dedicated personnel
☐ Challenges with vaccine acquisition
☐ Challenges with vaccine storage
☐ We frequently run out of vaccine due to limited stock
☐ Refer to other clinics
☐ HPV vaccines are controversial
☐ Not in our scope
☐ We choose not to
☐ Other

Please specify Other reason for not offering HPV vaccination.

At every patient encounter, do you assess HPV vaccination status?

◯ Never
◯ Sometimes
◯ Often/Always

For the unvaccinated, or incompletely vaccinated for HPV, do you recommend HPV vaccination?

◯ Never
◯ Sometimes
◯ Often/Always

How often do you recommend HPV vaccination to PARENTS when their child is:

	Never	Sometimes	Often/ Always
9-12 years old MALE	◯	◯	◯
9-12 years old FEMALE	◯	◯	◯
13-18 years old MALE	◯	◯	◯
13-18 years old FEMALE	◯	◯	◯

How often do you recommend HPV vaccination when an ADULT PATIENT (>18years) is:

	Never	Sometimes	Often/Always
18-26 year old MALE	◯	◯	◯
18-26 year old FEMALE	◯	◯	◯

Which of the following explains why you as an individual provider DO NOT OFTEN/ALWAYS assess, or recommend HPV vaccination?
(Select all that apply.)

☐ Not needed because we "bundle" vaccination
☐ I delegate these activities to other staff in my practice
☐ Additional training would be needed to perform some of these activities
☐ Not enough time
☐ Inadequate reimbursement
☐ Lack of effective tools and information to give to patients
☐ Patient can't afford the cost of the HPV vaccine
☐ I chose not to perform these activities
☐ Other

Please specify your Other reason for not performing the activities specified.

After you have recommended HPV vaccination, please indicate how often the vaccine is administered:

	Never	Rarely	Sometimes	Very often	Always	Not sure
if CHILD is 9-12 years old	◯	◯	◯	◯	◯	◯
if CHILD is 13-18 years old	◯	◯	◯	◯	◯	◯

if PATIENT is 18-26 years old ◯ ◯ ◯ ◯ ◯ ◯

Which reasons do PARENTS cite for NOT initiating HPV vaccination in their child?
(Select all that apply.)

- ☐ Not previously recommended
- ☐ Lack of knowledge or awareness of HPV vaccine
- ☐ Believe it is not needed
- ☐ Safety concerns / side effects
- ☐ Mistrust of government / health agencies
- ☐ Child is not sexually active
- ☐ Believe it promotes sexual activity
- ☐ Did not know it was recommended for males
- ☐ Religious or cultural beliefs
- ☐ Against HPV vaccination
- ☐ Out-of-pocket cost
- ☐ Other

Please specify the Other reason parents cite for not initiating HPV vaccination for their child.

Which reasons do your ADULT PATIENTS (>18 years) cite for NOT initiating HPV vaccination?
(Select all that apply.)

- ☐ Not previously recommended
- ☐ Lack of knowledge or awareness of HPV vaccine
- ☐ Believe it is not needed
- ☐ Safety concerns / side effects
- ☐ Mistrust of government / health agencies
- ☐ Not sexually active
- ☐ Did not know it was recommended for males
- ☐ Religious or cultural beliefs
- ☐ Against HPV vaccination
- ☐ Out-of-pocket cost
- ☐ Other

Please specify the Other reason patients cite for not initiating HPV vaccination.

Which reasons are cited by PARENTS for NOT completing recommended HPV vaccination series for their child/ren?
(Select all that apply.)

- ☐ Adverse reaction after 1st dose
- ☐ One dose was sufficient
- ☐ Competing priorities
- ☐ Transportation issues
- ☐ Pregnancy
- ☐ Out-of-pocket cost
- ☐ Other

Please specify the Other reason cited by parents for NOT completing a recommended HPV vaccination series for their child/ren?

Which reasons are cited by ADULT PATIENTS (>18 years) for NOT completing recommended HPV vaccination series?
(Select all that apply.)

☐ Adverse reaction after 1st dose
☐ One dose was sufficient
☐ Competing priorities
☐ Transportation issues
☐ Pregnancy
☐ Out-of-pocket cost
☐ Other

Please specify the other reason cited by patients for NOT completing a recommended HPV vaccination series?

How confident are you in your knowledge and ability to counsel PARENTS who are hesitant to vaccinate their child?

◯ Not at all
◯ Somewhat
◯ Moderate
◯ Very
◯ Completely

How confident are you in your knowledge and ability to counsel HPV vaccine-hesitant ADULT PATIENTS (>18 years) ?

◯ Not at all
◯ Somewhat
◯ Moderate
◯ Very
◯ Completely

After you have counseled HPV vaccine-hesitant PARENTS, please indicate the overall frequency at which they accept vaccination when their CHILD is 9-18 years old:

	Never	Rarely	Sometimes	Very often	Always	Not sure
After initial counseling session	◯	◯	◯	◯	◯	◯
Following subsequent counseling sessions	◯	◯	◯	◯	◯	◯

After you have counseled HPV vaccine-hesitant ADULT PATIENTS (>18 years old), please indicate the overall frequency at which they accept vaccination:

	Never	Rarely	Sometimes	Very often	Always	Not sure
After initial counseling session	◯	◯	◯	◯	◯	◯
Following subsequent counseling sessions	◯	◯	◯	◯	◯	◯

Which of the following is practiced at your facility?

	Yes	No	I don't know
Administer HPV vaccinations under standing orders	◯	◯	◯
Provide patients/parents a schedule of recommended HPV vaccinations	◯	◯	◯
Use reminders (e.g. phone or mail) when HPV vaccination is due	◯	◯	◯
Use recalls (e.g. computerized tracking) when HPV vaccination is past due	◯	◯	◯
Promote vaccination-related apps	◯	◯	◯
Bundling of HPV vaccination along with other vaccinations at the same visit (e.g. with meningococcal or Tdap)	◯	◯	◯

In your opinion, what is the single most important thing that can be done to increase HPV vaccination rates?

The next questions are about the impact of Coronavirus (Covid-19) pandemic on HPV vaccination.

During the Covid-19 pandemic, I have observed that

	Increased	Decreased	No change	Not sure
Intention to receive HPV vaccination	◯	◯	◯	◯
HPV vaccination hesitancy	◯	◯	◯	◯
HPV vaccination refusal	◯	◯	◯	◯
HPV vaccination acceptance	◯	◯	◯	◯
HPV vaccination uptake	◯	◯	◯	◯

What are some of the reasons given by patients/parents for the observed change(s) in HPV vaccination during the Covid-19 pandemic?
(Select all that apply)

☐ Increased mistrust of HPV vaccine
☐ Difficulties in access to HPV vaccination due to the pandemic
☐ Difficulties in transportation during the pandemic
☐ Difficulties in scheduling clinic visit during the pandemic
☐ Fear of contracting corona virus during the clinic visit

This section is about recent CDC guidelines for HPV vaccination

In August 2019, the CDC moved to recommend extension of HPV vaccination to include adults ages 27-45 years who are not adequately vaccinated and might be at risk for new HPV infection.

Before now, were you aware of this new recommendation?

◯ No
◯ Yes

According to the above CDC guidelines, providers are encouraged to employ shared decision making when recommending HPV vaccination to adults aged 27-45 years.

What, if any, barrier do you foresee in implementing this recommendation?

◯ No barriers, I plan to engage patients in shared decision making
◯ Time commitment
◯ I am not fully aware of the concept of shared decision making
◯ I am aware of shared decision making but unclear how to implement it for HPV vaccination
◯ Other barrier/s

Please specify Other barriers to implementation.

Demographics

How many years have you been in active medical practice?

____

On average, how many patients do you see per week?

____

What zip code do you primarily work in?

____

How old are you?

____

What is your sex?

◯ Female
◯ Male

Are you Hispanic, Latino/a origin?

◯ Yes
◯ No

What is your race?

◯ American Indian or Alaska Native
◯ Asian
◯ Black or African American
◯ White
◯ Other

Please indicate your Other race.

____

Thank you for your participation.

This completes questions regarding HPV vaccination.

We have developed resources for each of the cancer prevention topics listed below. We are looking for partners who can help implement strategies to tackle any or all of these areas of cancer prevention.
Are you willing to be a resource/advocate for any of the following cancer prevention areas?
(Select all that apply)

☐ Comprehensive tobacco control - to implement and disseminate evidence-based smoking cessation interventions
☐ Obesity/Energy balance - to disseminate innovative personalized behavioral interventions to aid in weight loss, dietary change, and physical activity
☐ Survivorship - to improve outcomes and well-being of cancer survivors in terms of quality of life, functioning and unmet needs
☐ Lung cancer screening and prevention - to deliver lung cancer screening among high-risk current or former smokers
☐ Breast cancer screening and prevention - to improve breast cancer risk-assessment, screening, and prevention
☐ Colon cancer screening and prevention - to improve colorectal cancer risk-assessment, screening, and prevention
☐ Cervical cancer screening and prevention - to improve cervical cancer risk-assessment, screening, and prevention
☐ Ultraviolet radiation exposure - to implement strategies for prevention and early detection of skin cancer
☐ Hepatocellular and renal cancers - to implement effective surveillance strategies for early diagnosis

Are you willing to be contacted for future studies?

◯ Yes
◯ No
